# Evaluation of a web-based registry of inherited bleeding disorders: a descriptive study of the Brazilian experience with *HEMOVIDAweb Coagulopatias*

**DOI:** 10.1186/s13023-016-0560-6

**Published:** 2017-02-10

**Authors:** Suely Meireles Rezende, Silvia Helena Lacerda Rodrigues, Kelly Neves Pinheiro Brito, Diego Lima Quintino da Silva, Marcos Lázaro Santo, Bárbara de Jesus Simões, Guilherme Genovez, Helder Teixeira Melo, João Paulo Baccara Araújo, Danila Augusta Accioly Varella Barca, on behalf of the HEMOVIDAweb Coagulopatias Study Group, Anelisa Schittini Costa Streva, Katty Pollyanni Ferreira Silveira, Sylvia Daniella Freitas de Souza, Sandra Sibele Leite Vieira de Figueiredo, Dario Itapary Nicolau, Irian Guedes Farkatt, Ana Elzira Dantas Leopoldino Rocha, Íris Maciel Costa, Rosângela de Albuquerque Ribeiro, Maria do Carmo da Silva Assunção, Viviane Pereira de Moraes, Ana Lice Sousa de Sena, Thereza Cristina Picado Pinheiro, Witânia Do Socorro Cardoso Silva, Pollyana Gomes de Souza Pimenta, Juliana Camila Lopes Cavaion, Julimara Moreira Rocha Leonel de Paiva, Dalva Gloria Ferreira França Barbacena, Sonia Maria Nunes De Barros, Maria do Perpétuo S. V. Orletti, Mitiko Murao, Luciene Figueiredo, Liana Andrade Labres de Souza, Denise Linhares Gerent, Giovana Fecker da Costa Diaz, Jouse Fonseca Bittencourt, Walquíria Lima de Almeida

**Affiliations:** 1 Department of Internal Medicine, Faculty of Medicine, Universidade Federal de Minas Gerais, Avenida Alfredo Balena, 190 – 2nd floor- room 243, Belo Horizonte, Minas Gerais ZIP 30130-110 Brazil; 2 Coordenação Geral de Sangue e Hemoderivados, Ministry of Health, SAF Sul, Edifício Premium, Torre II, room 202, CEP:70070-600 Brasília, Distrito Federal Brazil; 3 Departamento de Informática do Sistema Único de Saúde (DATASUS), Rua México - Centro, Rio de Janeiro, ZIP 20031-143 Brazil; 4 Hemocentro de Santa Catarina, Av. Othon Gama D’Eça, 756 Centro, Florianópolis, Santa Catarina ZIP 88015-240 Brazil; 5 DVB Consultoria em Gestão em Saúde, Brasília, Distrito Federal Brazil

**Keywords:** Bleeding, Haemophilia, von Willebrand disease, Registry, Brazil, Factor VIII, Factor IX

## Abstract

**Background:**

Inherited bleeding disorders (IBD) consist of a group of rare heterogeneous diseases, which require treatment for life. Management of these disorders is complex and costly. Therefore, good quality data of the affected population is crucial to guide policy planning. The aim of this manuscript is to describe the impact of a national, web-based registry – the Hemovidaweb Coagulopatias (HWC) – in the management of the IBD in Brazil.

**Methods:**

The system was developed in PHP 5.0 language and is available on the internet at http://coagulopatiasweb.datasus.gov.br. The system was validated in September 2008 and launched nationally with input from January 1, 2009. HWC collects variables related to socio-demographic, clinical, laboratory and treatment data of patients with IBD.

**Results:**

Within 7 years, there was an increment of 90.8% on the diagnosis of IBD altogether, which increased from 11,040 in December 2007 to 21,066 in December 2014. This is now the fourth and third largest world population of patients with haemophilia and von Willebrand’s disease (vWD), respectively, according to the most recent (2015) Annual Global Survey of the World Federation of Hemophilia. The data collected provided the basis for planning and implementing home therapy, prophylaxis and immune tolerance induction (ITI), recently initiated in Brazil.

**Conclusion:**

HWC was an effective tool in the increment of registration of patients with IBD in Brazil. Furthermore, it was essential to support policy planning, monitoring, evaluation and treatment. Future development should focus on surveillance, health outcomes and research. Every country should implement a national registry on IBD.

## Background

Inherited bleeding disorders (IBD) are heterogeneous group of rare diseases characterized by bleeding manifestations of variable severity. IBD require treatment for life, which is complex and costly. In most of the cases, treatment requires intravenous infusion of factor concentrates (FC) and/or blood products at prophylactic and/or episodic basis.

Clinical registries have a wide application in the management of IBD. Increasingly, national registries are providing patients, clinicians and governments an insight into the needs of patients, an important tool to guide health policies. Keeping updated registries is important for planning care, monitoring the effective use of resources, health outcomes and implementing surveillance [1]. Registries are particularly important for Government planning when a substantial amount of public resource is spent on groups of rare diseases, such as it is the case of the IBD.

Within the last years, there has been a substantial progress on the implementation of registries devoted to IBD in many countries [2–10]. However, many countries still do not have a national registry devoted to IBD [11] nor provide registry with good quality data [12]. In Brazil, until December 2008, the registration of patients with IBD was performed manually using spreadsheets, which had several limitations, such as difficulty in updating information, typing errors, duplications, and others. By noticing the need to improve data collection, the Ministry of Health (MoH), set up a modern, easily accessible web-based national registry of patients with IBD, the *HEMOVIDAWeb Coagulopatias* (HWC).

This report aims at describing the development and impact of HWC on registration, diagnosis and management of IBD in Brazil. To our concern this is the first report which shows an impact of a national web-based registry in comparison with a previous manually-collected registry.

## Methods

### Study

This is a descriptive study aimed at reporting the impact of a web-based registry of IBD in comparison with previous data manually-collected in *Excel* spread sheets [6]. Patients’ data were entered by health professionals working in the haemophilia treatment centers (HTCs).

### The program of IBD in Brazil

The MoH manages the Program of IBD in Brazil. The main roles of the Program are: (i) purchase of FC and distribution to HTCs, (ii) promotion of education of multiprofessional teams dealing with IBD, (iii) organization of guidances on management of IBD and (iv) provision of an updated registry.

The management and treatment of patients with IBD are guaranteed by the Brazilian Public National Health System – named *Sistema Único de Saúde* (SUS). Most patients with IBD are attended in HTCs located in the 26 Brazilian States and 1 Federal District. Patients who have private health insurance and milder forms of IBD are likely to be attended at private clinics. However, due to the fact that approximately 100% of FC purchased in Brazil is acquired centrally by the MoH, patients who demand treatment are attended at the HTCs. Therefore, it is reasonable to assume that approximately 100% of patients with moderate/severe forms of IBD are treated in the public sector (HTCs). For this, it is required that all patients are registered with a HTC in order to receive treatment.

### The *HEMOVIDAWeb Coagulopatias* system

The development of HWC started in early 2008 and was implemented from January 2009. Its development and management is funded by the MoH. It was triggered by the need of developing a national registry capable of collecting reliable clinical data of patients with IBD in the country and monitoring FC use.

The system was developed in PHP 5.0 language and is available on the internet at http://coagulopatiasweb.datasus.gov.br. The access to the system can be reached by using Internet Explorer 7, Mozilla Firefox or Google Chrome. The system was validated in September 2008 followed by a national training course for users in November 2008. It was then launched nationally with input from January 1, 2009.

The system was conceived by the creation of administrative modules assessed by individual username and password. Users signed a consent form which stated their responsibility to confidentiality and appropriate use of the system and data. The system contains six structured access profiles, classified as (i) Federal (MoH) Manager, (ii) State Manager, (iii) Care Provider, (iv) Service Provider, (v) Assistance Provider and (vi) Distribution Controller. Professionals in charge of these accesses are mainly haemophilia nurses, haemophilia doctors and pharmacists. The States are the responsible bodies for the provision of access profiles from ii to iv, in a decentralized flow under their decision.

### Modules

The modules were composed by two major groups: administrative and clinical. The administrative module was composed by: (i) registration, designed for registration of a new cases of IBD or transfer of a registered patient to another state; (ii) data extraction; (iii) emission of reports and (iv) stock control, use and distribution of FC.

The clinical module included: (i) socio-demographic data, (ii) diagnosis, (iii) clinical complications, (iv) laboratory tests and (v) treatment.

### Variables collected in the clinical module

Socio-demographic variables included: name, date of birth, sex, age, race, education, marital status, occupation, weight, height and body mass index.

The list of diagnosis comprised: HA or HB (if severe, moderate, mild or unknown), classification according to [13]; von Willebrand’s disease (vWD) (if types 1, 2A, 2B, 2 N, 2 M, 3, platelet type or unknown); afibrinogenaemia, hypofibrinogenaemia, dysfibrinogenemia, deficiencies of factors II, V, VII, X, XI, XII, XIII, high molecular weight kininogen, prekallikrein, PAI-1, combined deficiencies, deficiency of vitamin K-dependent factors, acquired inhibitors, Bernard-Soulier syndrome, Glanzmann’s thrombasthenia, other platelet disorders and other undiagnosed bleeding conditions. Variables on complications include: muscle skeletal complications; use of catheter; inhibitor status (screening and titration) and death (with causes).

Laboratory tests include: ABO blood group and Rh status; vaccination status for hepatitis A and B; serological tests for human immunodeficiency virus (HIV), human T lymphotropic virus (HTLV), hepatitis B and C. Whether any of these diseases are confirmed, we collect information regarding treatment.

Finally, in the treatment module, we collect: use of FC by type, data of infusion and quantity (in international units [IU] or other unit), lot, motive of use (surgery, trauma, bleeding, type of bleeding) and category of use (if home treatment, prophylaxis, episodic, hospital use, immune tolerance induction [ITI]). This module includes a tool for stock control, use and distribution of FC which is linked to MoH stock system.

## Results

### The impact of the system on the registration of patients and on the prevalence of IBD

**Fig. 1:**
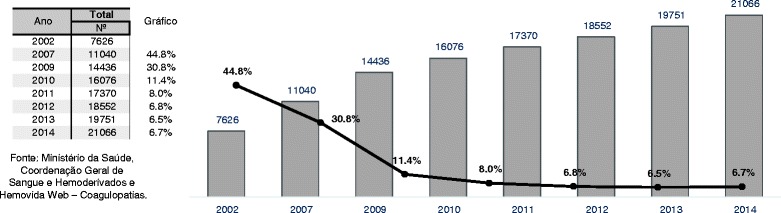
Prevalence of inherited bleeding disorders in Brazil, 2002–2014. The black line represents the increment percentage in the registration of patients from the previous year to the following year shown disclosed. There was a plateau from 2011 in the percentage of registration of new cases

In the first months after the implementation of the system, the number of registered patients with all forms of IBD increased 30.8% rising from 11,040 patients in December 2007 when the registrations were still performed in *Excel* spreadsheets to 14,436 patients in December 2009, the first year of use of HWC (Fig. 1). After 7 years, there was an increment of 90.8% in the registration of patients with all forms of IBD (from 11,040 in December 2007 to 21,066 in December 2014) (Fig. 1).

Following a sharp increase on the registration of cases from 2007 to 2010, we observe a tendency to a plateau from 2011, with a mean of 7% (variation, 6.5–8.0%) of new cases of IBD registered yearly from 2011–2014 (Fig. 1). This information is crucial for policy planning, acquisition of FC and estimation of the number of patients who will need prophylaxis and might develop inhibitors, according to reported incidences.

As expected, the prevalence coefficient of HA e HB also changed as a reflection of the increment in registration. The prevalence coefficient of HA increased from 0.73/10,000 in 2007 [6] to 1.00/10,000 males in 2014 [14]. For HB, the coefficient was 0.48/35,000 in 2007 [6] and 0.70/35,000 males in 2014 [14]. Differences between states remain, although less pronounced in 2014 when compared with 2007 (data not shown).

Historical data (2002–2014) on the relative distribution of the IBD showed a reduction in the percentage of HA and HB (in relation to all IBD) from 70.94 to 45.65% and 11.62 to 8.93%, respectively and an increase in the percentage of vWD and other IBD from 11.36 to 31.06% and 2.65 to 14.36%, respectively (Table 1). Rare bleeding disorders and platelet disorders account for the majority of the reported “other IBD” and their prevalence is detailed in Table 2.

**Table 1:** Historical prevalence of inherited bleeding disorders, Brazil, 2002-2014

Year	Haemophilia A		Haemophilia B		von Willebrand’s disease		Other inherited bleeding disorders^a^		Not informed		Total	
	N	%	N	N	N	%	N	%	N	%	N	%
2002	5,411	70.95	886	11.62	866	11.36	202	2.65	261	3.42	7,626	100
2007	6,881	62.33	1,291	11.69	2,333	21.13	316	2.86	219	1.98	11,040	100
2009	7,905	54.76	1,516	10.50	3,822	26.48	1,015	7.03	178	1.23	14,436	100
2010	8,369	52.06	1,609	10.01	4,451	27.69	1,437	8.94	210	1.31	16,076	100
2011	8,848	50.94	1,723	9.92	4,934	28.41	1,865	10.74	0	0.00	17,370	100
2012	9,122	49.17	1,801	9.71	5,445	29.35	2,184	11.77	0	0.00	18,552	100
2013	9,348	47.33	1,838	9.31	5,976	30.26	2,589	13.11	0	0.00	19,751	100
2014	9,616	45.65	1,881	8.93	6,544	31.06	3,025	14.36	0	0.00	21,066	100

^a^This includes rare bleeding disorders, platelet disorders, hemophilia carriers and unknown bleeding disorders

**Table 2:** Prevalence of rare bleeding and platelet disorders

Rare bleeding and platelet disorders	n	%
Afibrinogenemia	35	1.6
Hypofibrinogenemia	40	2.1
Dysfibrinogenemia	11	0.5
Factor II deficiency	13	0.6
Factor V deficiency	157	7.7
Combined factor V and VIII deficiency	27	1.1
Factor VII deficiency	723	35.4
Factor X deficiency	88	4.3
Factor XI deficiency	165	8.1
Factor XIII deficiency	61	3.1
Deficiency of vitamin K-dependent factors	13	0.6
Other combined deficiencies	46	2.3
Bernard Soulier syndrome	59	2.9
Glanzmann’s trombastenia	244	12.1
Other platelet disorders	359	17.6
Total	2,041	100

### The impact of the system on clinical data of IBD

In December 2014, most (62.6%) patients registered with IBD are female (after excluding all PWH) and 39% are in the age range of 20–39 years. In December 2014, severe, moderate and mild HA accounted for 38.3, 23.7 and 25.0% of patients, respectively. Severity was not informed in 13.0% of the patients, slightly lower than reported in 2007, 17.5% [6]. Severe, moderate and mild HB accounted for 31.4, 33.4 and 22.2%, respectively. Similar to HA, severity was not informed in 12.9% of the patients, slightly lower than reported in 2007, 14.2% [6].

In 2014, 77.8 and 79.0% of patients with HA and HB, respectively, were tested for inhibitors (screening test), when compared with 2007, which were 58.6 and 59.7%, respectively [6]. In 2014, 7.3% (*n* = 698) and 1.4% (*n* = 27) of patients with HA and HB, respectively, had a positive inhibitor screening test. Inhibitor titration was recorded in 77.4% (*n* = 540) of patients with HA, who had a positive screening test (*n* = 698). Inhibitors were of low-response (less than 5 BU/mL) in 43.7% (*n* = 236) and of high-response (>5 BU /mL) in 54.4% (*n* = 294) of patients with HA; in 10 patients (1.9%) titration was negative. Inhibitor titration was recorded in 70.3% (*n* = 19) of patients with HB who had a positive screening test (*n* = 27). Of these, 52.6% (*n* = 10) and 42.1% (*n* = 8) were low and high responders, respectively.

### The impact of the system on the assessment of factor concentrate use

The use of FC and other procoagulants (such as desmopressin acetate and tranexamic acid) are routinely recorded for all registered patients with IBD who receive treatment.

Regarding the categories of use of FVIII concentrate in 2014, secondary prophylaxis, home treatment, infusion in the HTC, ITI, hospital treatment and primary prophylaxis accounted for 49.9, 27.1,10.6, 8.0, 3.0 and 1.4%, respectively. For FIX concentrate, secondary prophylaxis, home treatment, infusion in the HTC, hospital treatment and primary prophylaxis accounted for 43.2, 29.0, 20.0, 6.8 and 1.0% of use in 2014, respectively.

When all PWHA (*n* = 9,616) and all PWHB (*n* = 1,881) were considered, mean use of FVIII and FIX concentrates per patient was 60,901 IU and 51,562 IU, respectively in 2014. However, 6,492/9,616 (67.5%) PWHA used FVIII concentrate in 2014, accounting for a (real) mean use of 90,207 IU of FVIII concentrate per patient. For PWHB, 1,250/1,875 (66.7%) used FIX concentrate in 2014, accounting for a (real) mean use of 77,343 IU per patient.

Furthermore, in 2014, 5.9, 24.1 and 65.2% of FVIII concentrate was used for the treatment of mild, moderate and severe PWHA, respectively. Patients with no information on the severity of HA used 4.8% of the totality of FVIII concentrate consumed in 2014. Regarding FIX concentrate, 8.1, 37.8 and 49.1% were used for the treatment of mild, moderate and severe PWHB, respectively. Patients with no information on the severity of HB used 5.0% of the totality of FIX concentrate consumed in 2014.

The increment of the purchase of FVIII and IX concentrates after 2010 in Brazil reflects the data on the per capita use of these products. In 2014, per capita use of FVIII and FIX in Brazil was 2.89 and 0.48, respectively [14]. This represents an increment of 136.9 and 84.6% when compared with data from 2010, which were 1.22 and 0.26, respectively [14]. Currently, 69.8 and 30.2% of PWHA are treated with recombinant and plasma-derived FVIII concentrate, respectively. The totality of PWHB are treated with plasma-derived FIX concentrate.

### The impact of the system on the implementation of prophylaxis and immune tolerance

Prophylaxis for severe and moderately-severe PWHA and PWHB (<2% of factor activity level) and ITI for PWHA and persistent inhibitors were implemented in Brazil in the end of 2011. Both programs were made available due to the provision of data on new cases of HA and HB, haemophilia severity and inhibitor development from January 2009. Since the registration of new cases is performed via web, in real time, upon inclusion of the PWH in the system and information on severity, it is possible to identify PWH who will require long-term prophylaxis. Furthermore, by identifying new patients with inhibitors it is possible to include them on ITI at an early stage. By December 2015, we had 383 PWHA and PWHB on primary prophylaxis and 240 patients with hemophilia A on ITI.

Concerning ITI, the first phase of the program included patients with long-standing history of inhibitors, selected from the database according to the inclusion criteria. More recently, as there is more awareness about ITI program and inhibitor surveillance increased, patients who develop inhibitor have been included earlier in the course of this complication. Furthermore, the system allows for searching candidate patients, therefore promoting a more efficient action on the identification of patients with inhibitors who fill the criteria for ITI.

## Discussion

The implementation of HWC, a web-based national registry had a major impact on the increment of the registration of patients with IBD, reflected by a rise of nearly 100% in the number of registered patients with all forms of IBD after 7 years of its onset. Furthermore, the system proved to be a good platform for the support of policy planning, promotion of health care, surveillance, allocation of resources and distribution of products.

According to the last published Annual Global Survey 2015 of the World Federation of Haemophilia (WFH), the total number of people with identified IBD in the world is 304,362 [15]. Within the 111 countries surveyed, Brazil has the fourth largest population of PWH (*n* = 11,857), after United States (*n* = 18,596), India (*n* = 16,635) and China (13,624) and the third population of patients with vWD (*n* = 7,223) after United States (*n* = 13,845) and United Kingdom (*n* = 10,586) [15]. This position was been achieved after the implementation of HWC*,* which promoted a sharp rise in the registration of patients with IBD.

There was a sharp increment on the prevalence of IBD in Brazil from 2002–2014 until 2010. After 2011, there was a plateau in the registration of “new” patients with IBD reaching about 7.0% a year. Therefore, in the first 3 years after its implementation, HWC was an efficient tool in the promotion of the registration of patients with IBD who were not already registered by the HTCs. However, since a major improvement in the diagnosis of these disorders occurred in Brazil in the same period, we cannot rule out the role of concurrent diagnostic strategies in the increment of the numbers registered.

The results here presented reflect patients’ data collected from the totality of the HTCs in Brazil, which represent of most of the patients with IBD in the country. Although we do not have the precise numbers, a small proportion of patients with mild IBD are attended in private clinics and therefore might not be registered with the HTCs. However, once these patients require treatment, they are referred to the public HTCs and are then registered in the HWC.

HWC had an important impact on the management of patients with IBD, reflected by the collection of reliable data as well as acting as a platform to policy planning. By assessing the prevalence of IBD and the mean use of products per disease and severity it was possible to estimate required budget and amount of FC needed to treat patients. Information regarding the real-time registration of new patients with hemophilia, its severity and new patients with inhibitors also supported implementation of prophylaxis and ITI. Both initiatives were implemented according to data generated by HWC. Furthermore, the distribution of FC for the entire country as well as its use is now easily monitored by the stock control tool, which allows the control of distribution and use of FC from the central stock at the MoH to the patient.

There was a prompt adherence of HWC by health professionals in the HTCs. This was likely due to its simplicity of use, easily accessible web-based registry, “real-time” provision of data and feasibility to download, save and print reports of the patients, which facilitated the incorporation of relevant medical information into patients’ files. Furthermore, we used a successful strategy for qualification and training of health professionals who worked with the system in the HTCs, which involved annual national meetings for discussion, presentation of data and innovations to the system. However, it is important to consider that part of the reason for a prompt adherence might be due to the compulsory need for registration of any patient with IBD who required use of any pro-coagulant product from January 2009.

HWC incorporated all the mandatory parameters recommended for the implementation of a national registry, such as personal and demographic, diagnosis and treatment details, bleeding history and serology [11, 16]. Furthermore, most of the optional parameters were also included such as joint status, death and cause of death, vaccination status and genetic information [11].

HWC has aspects which need improvement, either related to the (i) inclusion of additional parameters or to (ii) qualification of data. Additional parameters should focus on data related to hospitalization, surgery, quality of life assessment and genetic information. Qualification of data is still an important issue in many registries [12]. In HWC, we noticed that some variables such as vaccination status, serology, clinical data (severity of disease, inhibitor status, follow-up visits) and treatment data (infusion charts, detail of prophylactic scheme and adherence) are targets to improvement. Information on death and mortality cause is hardly obtained by HTCs. Furthermore, the registry does not pursue an appropriate strategy for surveillance parameters known to be important in IBD, such as inhibitor, infectious diseases, infusion reactions nor a facility which allow patients themselves to insert infusion data in the system.

## Conclusions

HWC, a national, web-based, real-time registry on IBD, had a major impact in the registration of IBD in Brazil in comparison with a manually-based national registry. Furthermore, its implementation promoted a prompt improvement on policy planning, which enhanced the quality of care of IBD in Brazil. We recommend all countries to implement national registries on IBD.

## Abbreviations

FC: Factor concentrate
HA: Hemophilia A
HB: Hemophilia B
HTC: Hemophilia treatment center
HWC: *Hemovidaweb Coagulopatias*
IBD: Inherited bleeding disorders
ITI: Immune tolerance induction
MoH: Ministry of health
PWHA: Patient with hemophilia A
PWHB: Patient with hemophilia B
SUS: *Sistema Unico de Saude*
vWD: von Willebrand disease
WFH: World federation of heophilia
